# Revisiting Polymorphic Diversity of Aminoglycoside *N*-Acetyltransferase AAC(6′)-Ib Based on Bacterial Genomes of Human, Animal, and Environmental Origins

**DOI:** 10.3389/fmicb.2018.01831

**Published:** 2018-08-10

**Authors:** Dae-Wi Kim, Cung N. Thawng, Kihyun Lee, Chang-Jun Cha

**Affiliations:** Department of Systems Biotechnology and Center for Antibiotic Resistome, Chung-Ang University, Anseong, South Korea

**Keywords:** AAC(6′)-Ib, polymorphism, genome database, One Health, epidemiology

## Abstract

The prevalence of *aac(6′)-Ib* variants has been demonstrated in numerous epidemiological studies. We revisited the polymorphic diversity of aminoglycoside 6’-*N*-acetyltransferase gene [*aac(6′)-Ib*] in the bacterial genome databases based on One Health perspectives. *aac(6′)-Ib* was searched against bacterial complete and draft genome databases of NCBI. Based on the major polymorphic residues 102, 117, and 179, taxonomy, ecology, and temporal emergence of bacterial isolates harboring variants of *aac(6′)-Ib* gene were evaluated using whole-genome sequences available in the databases. A total of 3,964 *aac(6′)-Ib* sequences were found to be present in the genomes of 34 bacterial genera, mostly found in Gammaproteobacteria. Among these, *aac(6′)-Ib-cr* variant, known to confer fluoroquinolone resistance, were increasingly detected in bacterial genomes and most abundant in the genera *Klebsiella* and *Escherichia*, thereby suggesting that these genera were the major reservoirs of the plasmid-mediated quinolone resistance (PMQR) determinant. The proportions of the cr variant were higher in animal and environmental isolates than in human isolates, among which the variant was dominant (>50%) in the genomes of intestinal, rectal, and fecal origins. In addition, our study suggested that the prevalence of the cr variant was associated with the occurrence of a variant with the mutation L117 (IbL). An integrated surveillance system for antimicrobial resistance in human, animal, and environmental sectors, based on whole-genome sequencing, would provide a better insight into the evolution, ecology, and epidemiology of antimicrobial-resistant bacteria.

## Introduction

The emergence and dissemination of antibiotic resistance cause a global public health crisis, a problem not only for humans, but also for animals and the environment, since the spread of resistant bacteria and resistance determinants appears to occur across human, animal, and environmental sectors (McEwen and Collignon, 2018). Recent studies demonstrated that animals and the environment are the major reservoirs of antibiotic resistance determinants, and hence, considered to be important routes for resistance dissemination (Mather et al., 2013; Perry and Wright, 2013; Forsberg et al., 2015; Woolhouse et al., 2015). Therefore, “One Health” approach, which encourages the integrative effort of multiple disciplines working locally, nationally, and globally to attain optimal health for people, animals, and the environment, is being emphasized to address this problem^1^ (Kahn, 2017a; Van Puyvelde et al., 2018). This approach, utilizing whole-genome sequencing surveillance in all three sectors, was proposed to be the gold standard to improve the understanding of evolution, ecology, and epidemiology of antimicrobial-resistant microbes (Allard, 2016; Kahn, 2017a,b).

Aminoglycoside *N*-acetyltransferase AAC(6′)-Ib was described in the 1980s and found to be broadly distributed in many clinically important isolates (Ramirez et al., 2013). Its variant AAC(6′)-Ib-cr, which harbors mutations at two amino acid residues (W102R and D179Y) and confers additional resistance against piperazine-containing fluoroquinolones, was first described in 2006 in the plasmids of clinical isolates of *Escherichia coli* isolated from Shanghai during 2000–2001 (Robicsek et al., 2006) and subsequently found to be widely disseminated around the world (Poirel et al., 2012; Jacoby et al., 2014). Earlier, another mutation (S117L) was reported to be associated with the altered resistance spectra against aminoglycosides (Casin et al., 1998). A variant harboring the mutation S117L conferred increased resistance to amikacin and reduced resistance to gentamicin, as shown by changes in the minimal inhibitory concentration values, from 2 to 16 mg/L for amikacin and from 4 to 0.25 mg/L for gentamicin (Rather et al., 1992). Recently, this mutation was found to be conserved in the cr variants of environmental origin, indicating the presence of three mutated residues in the cr variant (Kim et al., 2018). The cr variant is known to be usually located in a cassette as part of an integron in a multi-resistance plasmid, and considered as plasmid-mediated quinolone resistance (PMQR) (Jacoby et al., 2014). Numerous studies have been conducted for the epidemiology of wild-type and its variants using isolates of various origins (Park et al., 2006; Jiang et al., 2008; Yang et al., 2008; Kim et al., 2009; Sabtcheva et al., 2009; Frasson et al., 2011; Briales et al., 2012).

In our recent study, targeted gene sequencing analysis of *aac(6′)-Ib* gene from various DNA samples, including river, wastewater, chicken and human intestines, and soils, revealed that prominent polymorphisms occurred at the three mutated residues in the cr variant, and that this variant was dominant in soil and intestine samples, suggesting that the distribution of polymorphic variants was ecological niche-specific (Kim et al., 2018). Although this study was based on metagenomic DNA sequences and hence taxonomic information was absent, the results provided an overview of the prevalence of polymorphic variants at various ecological origins. In the present study, for more comprehensive understanding of the current status of *aac(6′)-Ib* prevalence, we explored taxonomy, ecology, and temporal emergence of bacterial isolates harboring variants of *aac(6′)-Ib* gene by extensive surveys of the bacterial genome databases.

## Materials and Methods

### Database and Search Criteria

The aminoglycoside *N*-acetyltransferase gene [*aac(6′)-Ib*] was searched against bacterial complete genome and draft genome databases of NCBI. *aac(6′)-Ib-cr* gene encoding fluoroquinolone acetylating aminoglycoside acetyltransferase (DQ303918.1) was used as a reference for BLASTn search against the databases. Sequence identity (>90%) and query coverage (>80%) were used as selection criteria for the gene.

### Polymorphism Analysis

*aac(6′)-Ib* gene sequences, searched from databases, were aligned with the reference gene and translated. After translation, Shannon entropy values (*H′*) and the relative abundance of polymorphic amino acids from residues 54 to 186 of AAC(6′)-Ib proteins were calculated as described previously (Iwai et al., 2010; Kim et al., 2018). Based on the major polymorphic residues 102, 117, and 179, all sequences were classified as follows: Ib (W102, S117, and D179), IbL (W102, L117, and D179), cr (R102, L117, and Y179), and others (other polymorphic variants) (Kim et al., 2018).

### Collection of BioSample Data

Information regarding various attributes such as isolation source, country, and collection year was obtained from BioSample accession numbers of bacterial genomes that contain *aac(6′)-Ib* gene (**Supplementary Table 1**). Although this information was not available in some cases, majority of genome datasets provided isolation source (81.6%), year (77.8%), and country (79.1%)-related information. Isolation sources of bacterial isolates were categorized as animal, environment, and human. Gene location was divided into chromosome or plasmid, based on the annotation of genome sequences.

### Phylogenetic Tree of Genera Harboring *aac(6′)-Ib* Gene

Phylogenetic tree was inferred by the maximum-likelihood method using type strains of type species of genera to which each bacterial genome belongs. Multiple sequence alignment was performed using MUSCLE v3.8 (Edgar, 2004). Alignment columns that contained >25% of end-gaps or >50% of internal gaps were trimmed. Nucleotide substitution model TIM3 with invariable sites and discrete Gamma models with four rate categories were selected, based on the model test performed by IQ-tree (Nguyen et al., 2015). A maximum likelihood phylogenetic tree was reconstructed using IQ-tree. The tree was rooted using a sequence of *Saccharibacteria* (KM462163) as an outgroup.

## Results and Discussion

### Polymorphic Variants of *aac(6′)-Ib* in the Bacterial Genome Databases

**FIGURE 1:**
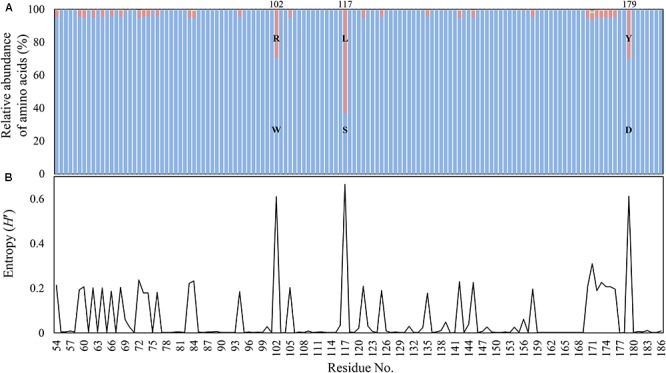
Polymorphism **(A)** and Shannon entropy **(B)** at 133 amino acid residues of AAC(6′)-Ib from the NCBI genome databases. Sequences showing >90% identity and >80% coverage with the reference protein (DQ303918) were selected as AAC(6′)-Ib and its variants. A total of 3,964 unique sequences were used for analysis. Blue bars indicate the proportion of amino acid sequences of wild-type (Ib) at each residue. Red, green, and purple bars indicate the proportion of polymorphic amino acids. Amino acids sequences of wild-type and major polymorphism at 102, 117, and 117 residues were displayed by single letter amino acid code.

Among >130,000 bacterial genomes available in March 2018 from the NCBI genome database, including both complete and draft genomes, a total of 3,964 *aac(6′)-Ib* gene sequences (370 and 3,594 from complete and draft genome databases, respectively) were found to be present in the bacterial genomes. The relative abundance and Shannon entropy analysis of polymorphism in the AAC(6′)-Ib proteins revealed that three residues (102, 117, and 179) were prominent polymorphic residues (**Figure 1**), as previously shown by the metagenomic studies conducted at various environmental sites (Kim et al., 2018). Composition of AAC(6′)-Ib variants (Ib, IbL, and cr) were highly similar in the complete and draft genome databases (**Supplementary Figure 1**). Analysis of gene location designated in the complete genome database revealed that IbL (89.3%) and cr (84.9%) variants were much more frequent in plasmids than in chromosomes (**Table 1**), whereas Ib (wild-type) sequences were similar among chromosomes and plasmids, thereby indicating that plasmids indeed form a major vehicle for the PMQR dissemination.

**Table 1:** Genetic location of *aac(6*′*)-Ib* and its variants reported in the NCBI complete genome database.

Location	Number of genomes containing each variant of *aac(6*′*)-Ib* (relative proportion, %)				
	Ib	IbL	cr	Others	Total
Chromosome	67 (52.3)	13 (10.7)	18 (15.1)	0	98
Plasmid	61 (47.7)	109 (89.3)	101 (84.9)	1	272


### Taxonomic Distribution of Polymorphic Variants of *aac(6′)-Ib*

**FIGURE 2:**
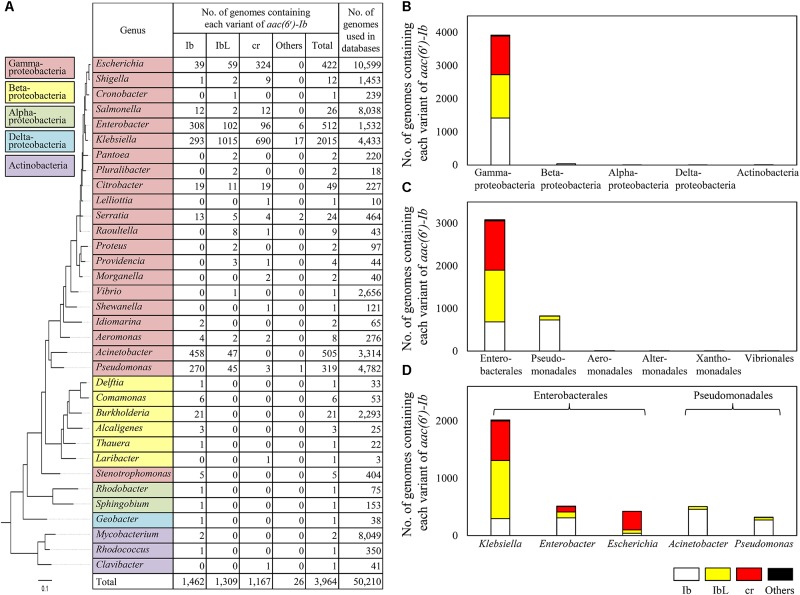
Taxonomic distribution of *aac(6′)-Ib* and its polymorphic variants in the NCBI genome databases. A maximum-likelihood phylogenetic tree was constructed based on the 16S rRNA gene sequences of type strain of type species to which bacterial genomes harboring *aac(6′)-Ib* gene belong. The number of genomes containing each variant of *aac(6′)-Ib* for each genus are shown in table **(A)** and presented in class **(B)**, order **(C)**, and genus **(D)** levels.

A total of 3,964 *aac(6*′*)-Ib* sequences were found to be present in the genomes of 34 bacterial genera (**Figure 2A**). The genes were mostly found in Gammaproteobacteria and rarely present in other bacterial taxa such as Alphaproteobacteria, Betaproteobacteria, Deltabacteria, and Actinobacteria (**Figure 2B**). Ten percent of the gammaproteobacterial genomes searched contained the gene. Among Gammaproteobacteria, a majority of *aac(6*′*)-Ib* genes were present in Enterobacterales and Pseudomonadales (**Figure 2C**). *Klebsiella*, *Enterobacter*, *Escherichia*, *Acinetobacter*, and *Pseudomonas* were the major genera harboring polymorphic variants of *aac(6*′*)-Ib* (**Figure 2D**). It is noteworthy that variants other than Ib, namely IbL and cr, were most abundant in the genera *Klebsiella* (69.9%), *Escherichia* (15.5%), and *Enterobacter* (8.0%), suggesting that these genera were the major reservoirs of those variants. In case of the cr variant, *Klebsiella* (59.1%) and *Escherichia* (27.8%) were the major taxa. The cr variant was absent or rare in Pseudomonadales, such as the genera *Acinetobacter* and *Pseudomonas*, where Ib was dominant (**Figure 2D**). The prevalence of wild-type Ib and the cr variant has been surveyed in various culture collections (Park et al., 2006; Jiang et al., 2008; Yang et al., 2008; Kim et al., 2009; Sabtcheva et al., 2009; Frasson et al., 2011; Briales et al., 2012). Most studies revealed that *Klebsiella*, *Escherichia*, *Enterobacter*, and *Citrobacter* belonging to *Enterobacteriaceae* were the major genera harboring *aac(6*′*)-Ib* gene, and the cr variant was highly prevalent in *Escherichia* and *Klebsiella* (Jacoby et al., 2014). These results were consistent with ours from the genome databases. Moreover, in this study, the presence of the cr variant was found to be correlated with the presence of IbL variant (*R*^2^ = 0.88) (**Supplementary Figure 2**), suggesting that the mutation at L117 might be a prerequisite for the emergence of the cr variant, and evolution of these variants might be associated with each other. Other polymorphic variants of *aac(6*′*)-Ib* such as R102/L117 and L117/Y179 variants, which were previously characterized to confer altered resistance spectra (Kim et al., 2018), were detected mainly in the genera *Klebsiella* and *Enterobacter* (**Supplementary Table 1**), indicating that these novel variants discovered in various environments were indeed present in clinical isolates.

### Ecological Distribution of Polymorphic Variants of *aac(6′)-Ib*

**FIGURE 3:**
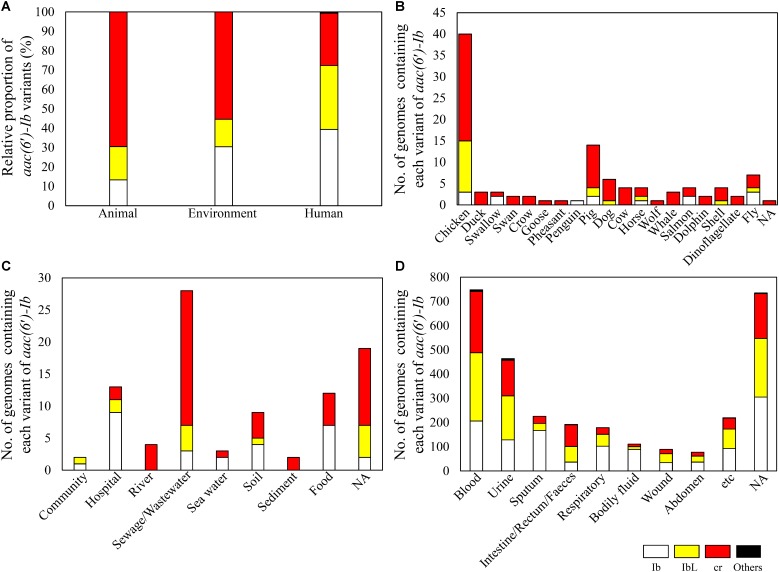
Prevalence of *aac(6′)-Ib* and its variants in the bacterial genomes of different ecological origins. The relative proportions of *aac(6′)-Ib* variants are shown in different ecological origins **(A)**. The number of genomes containing each variant of *aac(6′)-Ib* is presented according to detailed isolation sources of animal **(B)**, environment **(C)**, and human **(D)**.

When ecological information, obtained from the isolation sources of *aac(6′)-Ib-*containing bacterial genomes, was categorized as from animal, environment, and human sources, the three sectors showed different distribution of the *aac(6′)-Ib* polymorphic variants (**Figure 3A**). Animals and the environment displayed higher proportions of the cr variant than humans, where the variant was first discovered in the clinical isolate of *E. coli* (Robicsek et al., 2006). The cr variant was shown to be dominant regardless of animal type (**Figure 3B**). Among various environmental origins, the cr variant was dominant in sewage and wastewater (**Figure 3C**). Although Ib and IbL variants were shown to be more dominant than the cr variant in human isolates, the latter was dominant (>50%) in the bacterial genomes of intestinal, rectal, and fecal origins (**Figure 3D**). These results are consistent with the previous metagenomic study that revealed the cr variant as a major form in soil and intestine (chicken and human) microbiomes (Kim et al., 2018). Our current study, based on bacterial genomes, also suggests that sewage, wastewater, and human and animal microbiomes could be the major reservoirs of antibiotic resistance, as represented by *aac(6′)-Ib-cr*.

### Temporal Emergence and Geographical Distribution of Polymorphic Variants of *aac(6′)-Ib*

**FIGURE 4:**
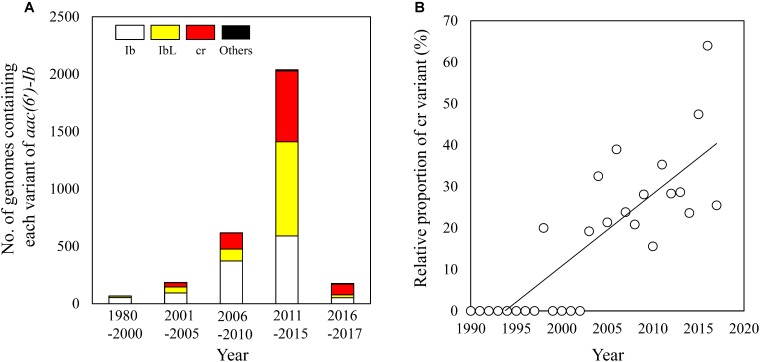
Temporal emergence of the cr variant reported in the NCBI genome databases. The number of genomes containing each variant of *aac(6′)-Ib* was summed for every five years since 1980 **(A)**. The relative proportions of the cr variant were plotted for each year since 1990 (*R*^2^ = 0.67) **(B)**.

With the increasing availability of bacterial genome sequence data, the number of genomes harboring *aac(6′)-Ib* gene has also been on the rise, although the number of such genomes isolated during 2016–2017 was much less deposited (**Figure 4A**). Notably, the cr variant of *aac(6′)-Ib* gene first appeared in the genome of *E. coli* strain MOD1-EC6136 isolated in 1983 (**Supplementary Table 1**; Gangiredla et al., 2017), although its first detection was reported in plasmids isolated during 2000–2001 (Robicsek et al., 2006). Furthermore, the proportion of the cr variants among all the *aac(6′)-Ib* variants found in bacterial genomes is increasing since its first discovery (**Figure 4B**). These results are consistent with the previous study which demonstrated the absence of the cr variants in isolates collected between 1981 and 1991 (Jacoby et al., 2009) but its prevalent since 1999 (Park et al., 2006), suggesting that the PMQR gene is still being disseminated.

The resistance gene has been detected in the genomes of isolates from 66 countries from all continents, being frequently found in United States, China, United Kingdom, Italy, South Africa, Brazil, India, Germany, Thailand, and Netherlands (**Supplementary Figure 3**). These results also indicate the global distribution of the gene.

## Conclusion

Previous epidemiological studies demonstrated the prevalence of *aac(6′)-Ib-cr* in many bacterial isolates. In the present study, we revisited the current status of taxonomy, ecology, and temporal emergence of *aac(6′)-Ib* variants using whole-genome sequences available in the public databases. Our results indicated that the cr variant was increasingly detected in bacterial genomes, *Klebsiella* and *Escherichia* being the major taxa harboring this gene. The proportions of the cr variant were higher in animal and environmental isolates than in human isolates, suggesting that animal and environment are reservoirs of the PMQR gene in the clinical settings. In addition, our study suggested that the prevalence of the cr variant was associated with the occurrence of IbL variant with the mutation L117.

Considering the importance of One Health approach, an integrated surveillance system for antimicrobial resistance in human, animal, and environmental sectors, based on whole-genome sequencing, would provide a better insight into evolution, ecology, and epidemiology of antimicrobial-resistant bacteria.

## Author Contributions

C-JC designed the research. D-WK and CT performed the analysis. KL conducted the phylogenetic analysis. D-WK and C-JC wrote the manuscript with contribution from all authors.

## Conflict of Interest Statement

The authors declare that the research was conducted in the absence of any commercial or financial relationships that could be construed as a potential conflict of interest.

## Footnotes

1 https://www.onehealthcommission.org/en/why_one_health/what_is_one_health/
