# Supplementary material for: Revisiting Polymorphic Diversity of Aminoglycoside N-Acetyltransferase AAC(6′)-Ib Based on Bacterial Genomes of Human, Animal, and Environmental Origins
Source: Front Microbiol. 2018 Aug 10;9:1831. doi: 10.3389/fmicb.2018.01831 (PMC6095969; doi:10.3389/fmicb.2018.01831)
Supplement: Supplementary file 1 [file Image_1.PDF]

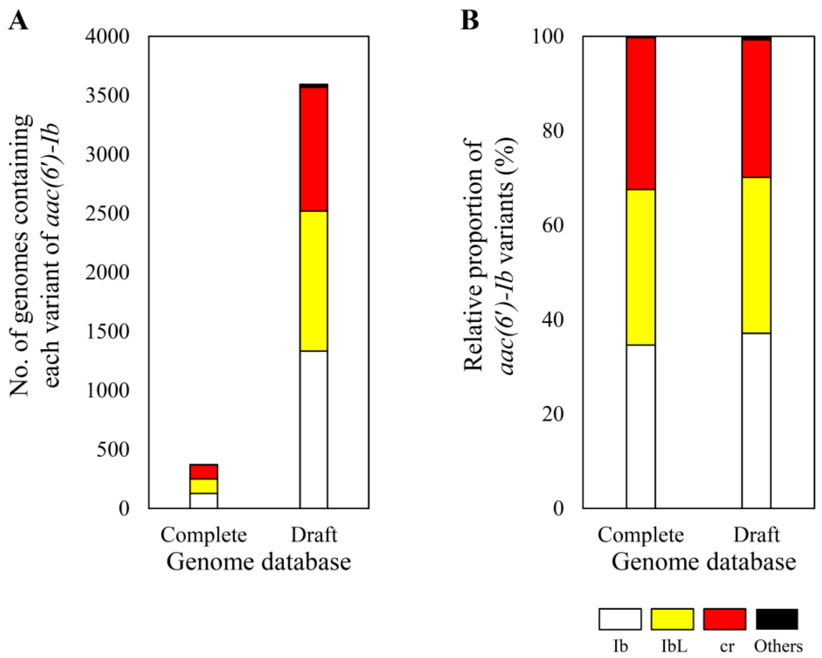

Supplementary Figure 1. Detection of *aac(6′)-Ib* gene from the NCBI complete and draft genome databases. The number of *aac(6′)-Ib* gene and its variants in each database is shown (a) and the relative proportions of each variant are displayed (b).
