# Supplementary material for: Revisiting Polymorphic Diversity of Aminoglycoside N-Acetyltransferase AAC(6′)-Ib Based on Bacterial Genomes of Human, Animal, and Environmental Origins
Source: Front Microbiol. 2018 Aug 10;9:1831. doi: 10.3389/fmicb.2018.01831 (PMC6095969; doi:10.3389/fmicb.2018.01831)
Supplement: Supplementary file 2 [file Image_2.PDF]

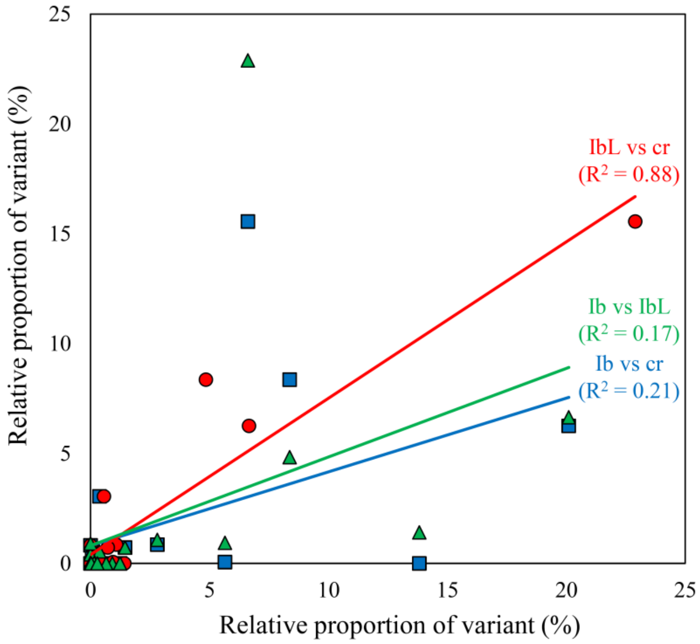

Supplementary Figure 2. Correlations between *aac(6′)-Ib* variants. Among the 34 genera harbouring *aac(6′)-Ib* gene in the NCBI genome databases, 19 genera with more than 100 deposited genomes were used. The relative proportions of two variants (Ib-IbL, Ib-cr, or IbL-cr) of the 19 genera were plotted and linear curve fitting between two variants was conducted.
