# Supplementary material for: Revisiting Polymorphic Diversity of Aminoglycoside N-Acetyltransferase AAC(6′)-Ib Based on Bacterial Genomes of Human, Animal, and Environmental Origins
Source: Front Microbiol. 2018 Aug 10;9:1831. doi: 10.3389/fmicb.2018.01831 (PMC6095969; doi:10.3389/fmicb.2018.01831)
Supplement: Supplementary file 3 [file Image_3.PDF]

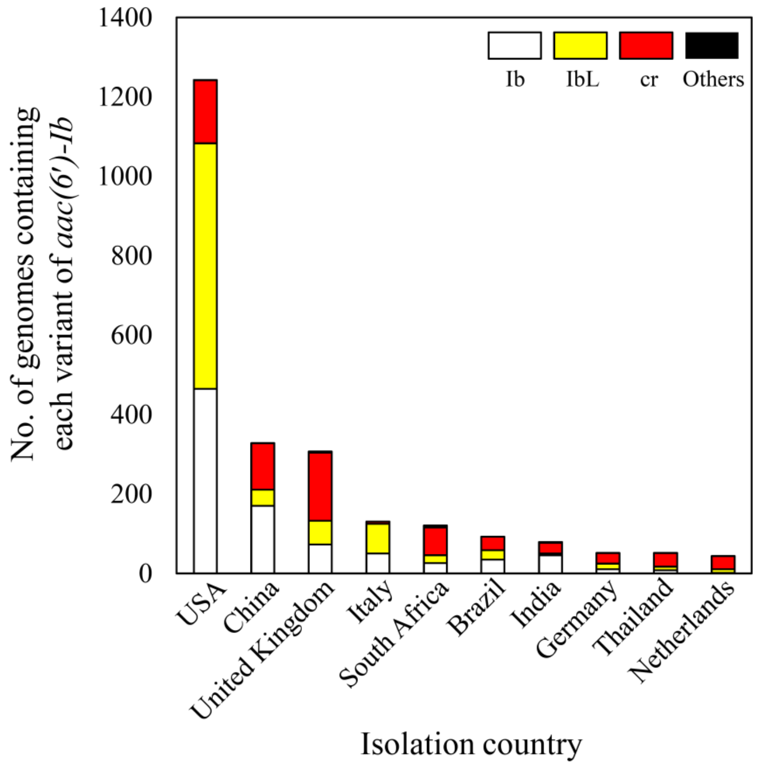

Supplementary Figure 3. Geographical distribution of *aac(6′)-Ib* gene and it variants in the NCBI genome databases. A total of 3,136 genomes were used. Among 66 countries, ten countries with the highest prevalence are displayed.
